# Trabecular Meshwork-Based MIGS: Efficacy, Technique Variability, and Wound Healing—A Comprehensive Review

**DOI:** 10.3390/jcm15041490

**Published:** 2026-02-13

**Authors:** Fahad R. Butt, Thanansayan Dhivagaran, Jacob Stasso, Kyran Sachdeva, Luckshann Arunasalam, Fatima Abid, Brendan K. Tao, Michael Balas, David J. Mathew

**Affiliations:** 1 Schulich School of Medicine & Dentistry, University of Western Ontario, London, ON N6A 5B8, Canada; fbutt2027@meds.uwo.ca (F.R.B.); tdhivagaran2027@meds.uwo.ca (T.D.); 2 Faculty of Medicine, University of Ottawa, Ottawa, ON K1H 8M5, Canada; jstas083@uottawa.ca (J.S.); ksach080@uottawa.ca (K.S.); 3 Faculty of Science, McMaster University, Hamilton, ON L8S 4K1, Canada; arunasal@mcmaster.ca; 4 Faculty of Science, Toronto Metropolitan University, Toronto, ON M5B 2K3, Canada; fatima.abid@torontomu.ca; 5 Department of Ophthalmology & Vision Sciences, University of Toronto, Toronto, ON M5T 3A9, Canada

**Keywords:** MIGS, trabecular meshwork, review, glaucoma

## Abstract

**Background:** Glaucoma, a leading cause of blindness worldwide, can be managed surgically when medical treatments are insufficient. Trabecular meshwork (TM)-based micro-invasive glaucoma surgery (MIGS) has emerged as a less invasive surgical approach to lowering intraocular pressure (IOP) and managing glaucoma. **Methods:** This narrative review synthesizes the scientific literature and trials on TM-based MIGS techniques, including trabecular micro-bypass stents, goniotomy and trabeculotomy procedures, electrocautery and laser-based approaches, and ab interno canaloplasty. Among these techniques, efficacy, pivotal clinical trials, medication reduction, safety profiles, complications, and determinants of surgical success were examined. **Results:** While all TM-based MIGS devices assessed achieved meaningful IOP reduction compared with trabeculectomies, there was no consensus on which TM-based MIGS technique yielded the best outcomes. TM-based MIGS had a good safety profile compared with traditional glaucoma surgeries. Common postoperative complications included transient hyphema, corneal edema, and anterior chamber inflammation. Ultimately, the success of TM-based MIGS procedures depended on many factors, including surgical technique, device type, and patient-specific factors. **Conclusions:** TM-based MIGS are an effective and safe surgical management option for eligible glaucoma patients requiring moderate IOP reduction. Future research should focus on the long-term outcomes of MIGS procedures, compare MIS devices, and explore the mechanistic and histopathological changes induced by MIGS.

## 1. Introduction

Glaucoma is one of the leading causes of blindness, affecting 76 million people globally, with an estimated 111.8 million predicted cases by 2040 [1]. Glaucoma is a progressive form of optic neuropathy that eventually causes irreversible vision loss when left untreated [1]. The pathophysiology of glaucoma typically involves reduced outflow, leading to an increase in intraocular pressure (IOP) [2]. Chronic IOP elevation places mechanical stress on the optic nerve head, resulting in disrupted blood flow, retinal nerve fiber functional impairment, and eventually retinal ganglion cell (RGC) death [3].

Non-modifiable risk factors for glaucoma include age, family history, and ethnicity [4,5]. Modifiable risk factors other than IOP, such as obstructive sleep apnea, diabetes, and hypertension, can also contribute to progression [6,7,8]. While some forms of glaucoma do not exhibit increased IOP (i.e., normal-tension glaucoma), IOP lowering remains the primary target for glaucoma management [4,9].

The ultimate goal in glaucoma management is to maintain nerve health and visual function while preventing disease onset or progression [10]. Treatment strategies can vary but generally follow a stepwise approach to IOP reduction, starting with either pharmacotherapy or laser trabeculoplasty [3,9,11]. For treatment-resistant cases or severe glaucoma, trabeculectomy and glaucoma drainage device insertion are considered, although these are more invasive procedures with elevated risk of complications [12,13,14,15]. This limitation prompted the development of minimally invasive glaucoma surgery (MIGS) for cases resistant to mild-case management, but not severe enough for surgical intervention [16].

MIGS provides a less invasive means to lower IOP in mild-to-moderate glaucoma otherwise resistant to pharmacotherapy and laser therapy [16,17], while preserving internal anatomical integrity, involving fewer complications, and allowing for quicker recovery [16]. However, MIGS also has shortcomings, typically achieving only moderate IOP reduction and complication profiles specific to device design that could impact long-term efficacy [17,18]. Furthermore, another drawback is the novelty of MIGS and the lack of long-term outcomes for newer MIGS devices [17,18,19].

MIGS procedures target anatomical structures playing key roles in IOP regulation, such as the trabecular meshwork (TM) [2]. Non-TM MIGS focuses on either providing alternate pathways of aqueous flow, such as suprachoroidal micro-stenting, or reducing total aqueous fluid production, through ciliary body ablation [18,20]. Minimally invasive bleb surgery (MIBS) lowers IOP through the creation of a subconjunctival filtration bleb using microstents or microshunts [12,20]. Examples of MIBS devices include the XEN Gel Stent, which is an ab interno implant, and the PreserFlo MicroShunt, which is an ab externo device [12,20]. However, the focus in this review is on TM-based MIGS, which is designed to overcome aqueous outflow resistance at the primary site of pathology, the juxtacanalicular TM [2]. By bypassing or removing TM tissue, these procedures increase outflow through Schlemm’s canal and effectively lower IOP [21]. They are generally indicated for mild-to-moderate open-angle glaucoma (OAG) in patients who need better IOP control or are already undergoing cataract surgery [16,21]. This review will provide an in-depth perspective on TM MIGS approaches and their role in glaucoma care, examining the efficacy, safety-profile, and post-operative outcomes of these devices.

## 2. Review of TM MIGS Techniques

All TM MIGS procedures are performed using an ab interno (inside out) approach beginning with a temporal clear corneal incision (~1.5–2.0 mm) [16]. The anterior chamber (AC) is deepened using an ophthalmic viscoelastic device (OVD) to maintain space and protect nearby intraocular structures [16]. Intraoperative gonioscopy is performed to visualize and identify the TM. These steps provide access to Schlemm’s canal for device placement and TM manipulation [16]. Post-operative treatment involves steroids and glaucoma drops as needed [16]. TM-based MIGS approaches are summarized in Table 1.

### 2.1. iStent/IStent Inject

The iStent and iStent Inject systems are TM micro-bypass stents designed to create a focal conduit from the AC into Schlemm’s canal [22]. The first-generation iStent is a single heparin-coated titanium implant inserted into the TM and inner wall of Schlemm’s canal via gentle forward pressure [23,24]. Once the stent’s lumen is within the canal, the stent is slightly advanced and released from the inserter [22]. The iStent injector releases two smaller stents, whereby the first is released into the canal followed by rotation of the injector prior to implantation of the second stent [22]. The injector is then removed and the gonioprism is used to confirm correct positioning within Schlemm’s canal [16,25]. Stents avoid broad damage to the TM, facilitating less immediate blood reflux, but if the stent ostium or distal channels scar, the effect may be diminished [26].

### 2.2. Hydrus Microstent

The Hydrus Microstent is a nitinol scaffold that is designed to bypass the TM and dilate Schlemm’s canal over approximately 90° [27]. It is preloaded and delivered through a stainless-steel cannula, which is passed through the TM into Schlemm’s canal [27]. The delivery wheel is rolled to slightly advance the microstent within Schlemm’s canal with correct placement verified by visual inspection [27]. Unlike the iStent, Hydrus scaffolds a larger canal segment, facilitating extended access to distal collector channels [27]. However, like other stents, its efficacy can diminish if distal resistance persists [28].

### 2.3. Trabectome (Ab Interno Electrocautery Trabeculectomy)

The Trabectome handpiece is connected to the Trabectome console [29]. The Trabectome device emits high-frequency energy (550 kHz plasma), ablating TM tissue upon contact [30]. During cauterization, the handpiece must be slowly swept along the angle (typically 60–120° arc) to remove a continuous strip of TM while the aspiration port removes debris to maintain a clear view [30]. Blood reflux from collector channels, filling the canal or anterior chamber, is common once the TM is removed, indicating communication with Schlemm’s canal, often presenting as mild hyphema [30]. The incision is then hydrated to seal and sutures are typically not necessary [30]. As a form of thermal ablation, Trabectome removes resistance more broadly than focal stents [30]. It provides hemostatic stability but may cause visual disturbance from the cautery plume [30]. Studies show it can achieve greater outflow per treated area and one study reported higher success than KDB at 12 months (54% vs. 30%), using IOP <21 mmHg and ≥20% reduction as criteria [28].

### 2.4. Kahook Dual Blade (Ab Interno Goniotomy)

In Kahook Dual Blade (KDB) goniotomy, TM tissue is straddled and caught by a dual blade, allowing entrance into Schlemm’s canal following advancement of the blade [31]. The dual blades create parallel incisions, effectively lifting and cutting a strip of TM tissue as the device is advanced 3–4 clock hours along the angle, which may be removed by micro-forceps or aspirated [31]. KDB achieves mechanically clean TM excision without capital equipment, potentially reducing fibrosis risk [31]. However, it typically causes more immediate blood reflux than the Trabectome [28].

### 2.5. TRAB360 Device (360° Trabeculotomy with Microcatheter)

TRAB360 offers a circumferential trabeculectomy by threading a flexible microcatheter filament into Schlemm’s canal [32]. The microcatheter is steadily retracted upon completion of a loop or until resisted, which tears the TM and results in a 180° or 360° trabeculotomy [32]. Postoperatively, a greater degree of hyphema is expected compared to focal goniotomy due to the large area of Schlemm’s canal opened [33]. Circumferential trabeculotomy such as TRAB360 and gonioscopy-assisted transluminal trabeculotomy (GATT) provides greater IOP reduction, particularly in eyes with higher baseline pressures [33]. These procedures expose all collector channel ostia, improving outflow but increasing the risk of early IOP spikes and hyphema [32,34,35,36].

### 2.6. GATT (Suture-Based 360° Trabeculotomy Ab Interno)

In GATT, a blunted 5-0 or 6-0 polypropylene suture is inserted into Schlemm’s canal through goniotomy, or alternatively, an illuminated microcatheter may be used [16,30,31]. Using micro-forceps, the suture is fed around the circumference of the canal and advanced stepwise [32]. The 360° suture is then threaded until its tip emerges from the opposing end of the canal, either back at the goniotomy or through a second small incision [32,37]. If a continuous suture is used, both ends of the suture will be externalized in the AC, forming a loop through the canal [32,37]. If a microcatheter is used, a suture is tied to the catheter tip, and one end of the suture is pulled while the other end is held stable to tear the TM [32,37,38]. As the suture slides through the canal, the TM is unroofed, resulting in a full 360° trabeculotomy [16,32].

### 2.7. Excimer Laser Trabeculostomy (ELT)

ELT setup requires a specialized excimer laser console with a handheld fiber-optic probe that is inserted into the AC and approaches the TM [39]. The tip is placed at the target location on the TM, ensuring it is flush without indenting too deeply [39]. The excimer laser is activated to deliver short nanosecond pulses of 308 nm energy through the probe onto the TM [40]. Typically, several spaced microperforations are made adjacently along an arc of ~90° of the angle, preventing excessive weakening of the structural integrity while providing multiple outflow channels [39]. There is no tissue to remove since the laser ablates it into plasma, and as such, debris is minimal [39]. Typically, ELT has a very mild postoperative course, with minimal inflammation and rare hyphema [41]. ELT may be preferable in anticoagulated patients or those in whom tissue manipulation poses higher risk [42]. However, it is less commonly performed globally due to equipment requirements, and its effect on outflow is modest [43].

### 2.8. Ab Interno Canaloplasty (ABiC)

ABiC involves the insertion of a flexible iTrack microcatheter into Schlemm’s canal via the goniotomy [44]. A catheter with illuminated tip to track progress is threaded around the full 360° of Schlemm’s canal if possible, though at least 180° in each direction from the two incisions can be used as an alternative [44]. Unlike goniotomy or trabeculotomy, ABiC leaves the TM intact except for the initial small goniotomy. As such, intraoperative blood reflux is usually minimal, though viscoelastic may push blood through the distal channels [44]. ABiC provides modest IOP reduction, often a few mmHg more than cataract surgery alone [45]. It is ideal for early glaucoma or ocular hypertension, offering a favorable safety profile with low risk of hyphema or hypotony [46]. However, it generally does not achieve the same IOP lowering as full-thickness TM procedures, especially if TM remains a site of resistance after viscodilation [45].

### 2.9. Ab Interno Needle Goniectomy (BANG)

Ab interno needle goniectomy (BANG) is a low-cost TM MIGS procedure that excises trabecular meshwork with a hypodermic needle in order to improve aqueous outflow and lower IOP [47]. Dada et al. (2023) describe the procedure for performing BANG [48]. The 30-gauge needle, which is attached to a syringe filled with cohesive viscoelastic, is bent at the proximal junction of the bevel [48]. The needle is introduced ab interno through a clear corneal incision and placed into Schlemm’s canal via the trabecular meshwork, where the viscoelastic is injected in order to dilate Schlemm’s canal [48]. Subsequently, the trabecular meshwork strip is excised, creating direct communication between the anterior chamber and Schlemm’s canal to improve aqueous outflow and lower IOP [48]. A recent interventional study including 30 eyes reported a mean IOP decrease from 15.3 to 13.57 mmHg (*p* = 0.028) six months following BANG [49]. Additionally, a retrospective chart review of 43 eyes of patients with POAG who were treated with BANG with cataract surgery reported an average IOP reduction of 20.80% at 3 months [50]. Another study found that when BANG was performed on 41 eyes with open-angle glaucoma (mild to severe), there was at least a 20% decrease in IOP in 73% of patients, and 41% had an IOP of less than 12 mmHg [47]. One of the main complications is damage to angle structures and the outer wall of Schlemm’s canal [48]. While transient hyphema can occur following BANG, studies do not report significant loss in visual acuity 1-year post-BANG [49]. Visual field outcomes following BANG remain stable [51]. A prospective interventional study including 32 eyes with POAG reported stable visual field mean deviation at 12 months [51].

## 3. Mechanisms of IOP Reduction and Wound Healing

All TM MIGS ultimately lower IOP by decreasing the resistance to aqueous humor outflow through the TM to episcleral veins [52]. While MIGS approaches promote conventional outflow, they typically cannot achieve extremely low IOPs since they do not bypass the episcleral venous pressure (EVP), usually ~8–10 mmHg [52]. Thus, MIGS is particularly useful in cases where moderate IOP reduction is sufficient, often in earlier stages of glaucoma, while reductions are usually insufficient in severe glaucoma [53]. For example, at ~7.5 years post-op, Trabectome patients had a 29% IOP reduction (23 → 16.5 mmHg), whereas trabeculectomy usually yields ~40–50% reductions (e.g., 24 → 12 mmHg) in a similar timeframe [54,55].

### 3.1. Wound Healing and Tissue Response in TM MIGS

A major advantage of MIGS is the reduced wound healing response compared to filtering surgery [16]. Since MIGS approaches are internal and microincisional, they spare the conjunctiva and typically induce less inflammation [16]. However, at the microscopic level in the angle, wound healing processes still occur and can influence long-term success [16].

### 3.2. Stents (iStent/Hydrus) Tissue Response

Histopathologic studies in human eyes with iStents have shown a benign tissue response: the stent lumen remains open, and surrounding TM might atrophy slightly from disuse, but there is generally no significant fibrous encapsulation around the device [56]. The heparin coating and constant aqueous flow likely deter fibrosis/clot formation [57]. In Hydrus, five-year clinical data indicate stable results with no evidence of progressive occlusion [27]. Gonioscopic and optical coherence tomography (OCT) imaging of Hydrus implants show that the canal remains dilated around the device, and fibrocellular reaction is minimal [27]. One area of wound healing concern is the formation of a fibrin plug early after implantation, although this usually resolves with time [30]. However, unlike subconjunctival filtering blebs, these internal stents do not elicit Tenon’s capsule fibrosis or scarring that progressively constricts flow [58,59,60].

### 3.3. Goniotomy/Trabeculotomy Healing

When TM is excised or incised, the body will attempt to heal that opening. However, unlike a skin wound, the continuous flow of aqueous and lack of exposed conjunctival tissue makes the healing response less aggressive [61]. After excisional KDB goniotomy, the removed strip of TM is gone permanently [62]. The edges of the remaining TM might curl outward a bit [62]. Over weeks, a membrane can form over the cleft which is believed to be composed of endothelial cells and fibrin, possibly creeping from the residual TM or from the inner wall remnants [63]. Histology from some goniotomy specimens shows thin neomembrane formation covering part of the cleft, which could increase outflow resistance again [63]. The KDB’s parallel incisions ideally remove the entire TM strip cleanly, minimizing residual leaflets, which may lead to less fibrosis within the cleft [30,62]. Indeed, anecdotally and in gonioscopic follow-up, KDB-created clefts tend to remain visibly open months later, whereas a simple slit goniotomy might show reconnected tissue bridges [30,62].

For Trabectome (electrocautery), the thermal ablation may confer some advantage by cauterizing cut edges, potentially reducing bleeding and perhaps scarring [64]. Some evidence suggests the TM does not significantly regenerate after Trabectome ablation [65]. Histologically, minimal damage to the posterior wall of Schlemm’s canal and surrounding tissues may be seen [66,67].

### 3.4. 360° Trabeculotomy (GATT) Healing

As the entire TM circumference is disrupted during GATT, the healing response is diffuse [38]. A 360° trabeculotomy in congenital glaucoma can lead to a re-scarring of some regions, particularly if done very early in life, since the elastic TM can fuse back in parts [38]. In adult eyes, once the TM is torn, it is less likely to rejoin fully [68]. Throughout healing, some segments of the canal opening may be covered by a fine layer of fibrous tissue [68]. However, the circumference usually remains sufficiently patent to maintain lowered IOP [68]. The high success rates of GATT in case series of 103 eyes from 84 patients at 1–2 years suggest that significant re-scarring is not common [68].

### 3.5. Wound Modulation

In contrast to trabeculectomy, antifibrotic agents (e.g., MMC) are not used during TM-based MIGS, since excessive wound healing is not expected and applying such agents may risk endothelial damage [69]. The natural environment of the aqueous humor, consisting primarily of water and low protein content, diminishes its capacity for a strong fibrotic response [12]. In particular, low protein content restricts fibroblast activation and extracellular matrix deposition, both of which are key mediators of the fibrotic response [12]. Moreover, any fibrosis that does occur in MIGS affects specific structures such as Schlemm’s canal or the TM, which are less prone to sight-threatening fibrosis [16].

### 3.6. Bleeding

Mild bleeding that occurs in trabecular MIGS as blood reflux may indicate a patent Schlemm’s canal and collector channels, which is generally considered a favorable sign. In most cases, the resulting hyphema is self-limiting, aided by aqueous turnover and, when necessary, fibrinolytic processes [70].

### 3.7. Histopathological Evidence

A preclinical histology study by Ammar et al. (2020) compared goniotomy techniques (including KDB, Trabectome, etc.) in porcine eyes [71]. It found that the KDB excisional goniotomy resulted in the least residual TM tags and a more open Schlemm’s canal, whereas straight incision left TM flaps that could collapse [71]. Another study examining tissue obtained from KDB goniotomy in humans showed that the excised TM strips were intact enough to analyze, indicating the precision with which tissues can be removed [63]. The histological findings indicate that KDB goniotomy tissue had a higher yield of TM compared to traditional ab externo trabeculectomy. This suggests that tissue injury from KDB goniotomy is confined to the TM, which supports the procedure’s predictable healing profile [63].

## 4. Indications and Patient Selection

Different MIGS techniques may be preferred based on patient factors [72]. For instance, if a patient has mild OAG, and especially if a cataract is present, implanting an iStent inject or Hydrus with cataract surgery is a convenient choice supported by FDA approval and large trials [72]. These stents work well when moderate IOP reduction is adequate, and prompt recovery is valuable [72]. By contrast, an eye with more advanced glaucoma may require a combination of multiple MIGS techniques or choice of a more extensive outflow procedure like goniotomy or trabeculotomy procedures for necessary IOP reduction [31,73,74]. In patients with healthier distal outflow pathways, often younger individuals (less than 60 years old) or those with secondary glaucomas, such as pigmentary glaucoma and pseudoexfoliation (PXF) glaucoma, an excisional MIGS procedure removing pigment deposits and heavily clogged TM such as KDB or Trabectome may be more suitable for IOP reduction, as distal outflow would be relatively undisturbed [31,73,74]. PXF glaucoma may respond better to trabecular bypass surgery than primary OAG [75].

In congenital or juvenile glaucoma, GATT or 360° trabeculotomy are often selected [76,77]. GATT enables an internal approach, unlike traditional 360° trabeculotomy, which is the current gold standard for primary congenital glaucoma [76,77]. Trabecular micro-bypass stents are rarely used in pediatric glaucoma due to limited evidence of efficacy, unlike goniotomy and trabeculotomy, which are well-supported by the existing literature [76,77]. MIGS devices such as the iStent and Hydrus lack systematic evaluation in children and may be constrained by anatomical variability, surgical access, and long-term durability concerns [78,79,80,81].

If a patient is pseudophakic or not planning cataract surgery, standalone MIGS choices are applicable [82]. In the US, iStent was initially approved only for use with cataract surgery, steering surgeons toward goniotomy-based MIGS for standalone cases [83]. For example, an older pseudophakic patient on two medications with moderate glaucoma might benefit from a KDB goniotomy or an OMNI (viscodilation + trabeculotomy) to delay or avoid a trabeculectomy [83]. Now, newer devices like the iStent infinite (three stents) are being approved for standalone use, expanding options [83]. In general, however, evidence suggests that MIGS has better outcomes when combined with phacoemulsification due to its IOP-lowering effect and potentially improved outflow post-lensectomy [83,84]. For instance, a systematic review included 279 articles found that cataract surgery combined with a trabecular procedures demonstrated an added 1.6 to 2.3 mmHg IOP reduction compared to only cataract surgery [85].

## 5. Outcomes and Efficacy

All included TM MIGS devices achieve meaningful IOP reduction while providing a better safety profile compared to traditional glaucoma surgeries such as trabeculectomies [32,86]. Differences in efficacy of MIGS devices for IOP reduction are summarized in Table 2. With regard to IOP reduction, MIGS procedures that favored removal or bypassed significant portions of the TM achieved greater drops in IOP. For instance, 360° trabeculotomy with Trab360 or GATT devices, and multiple stents with the Hydrus Microstent, are associated with significant IOP reductions [32,86]. For example, a retrospective case series found that the Hydrus Microstent produced a mean IOP reduction of 26.7% in 101 eyes [32,86]. IOP reduction with MIGS helps stabilize visual fields, with long-term data demonstrating slower VF deterioration in bypass stents compared to sole reliance on pharmaceutical treatment [87]. A 7-year German study of iStent inject in 125 adult eyes with open-angle glaucoma found that only ~4.8% of eyes had clinically significant VF deterioration (defined as VF MD loss ≥ 2.5 dB over 7 years post-op) [32,88]. In contrast, historical data suggest that, without adequate treatment, around 50 to 60% of glaucoma patients may experience disease progression over a 7-year period [21,88]. Smaller case series of ab interno canaloplasty and GATT also report that the vast majority of patients have unchanged visual field indices postoperatively, especially when high baseline IOP is lowered substantially [45,89]. Overall, the TM MIGS devices are minimally invasive and do not compromise visual acuity, as measured by VF [45,89]. Often, when conducted in conjunction with cataract surgery, there is overall improvement in visual acuity [45,89].

Head-to-head comparisons are limited, but available data highlight differences. Notably, in a prospective randomized trial, an iStent inject resulted in greater IOP reduction than a single first-generation iStent: mean IOP drop was 26.6% with iStent inject versus 15.8% with iStent at 6 months with similar safety profiles [90]. This aligns with other studies showing that multiple trabecular micro-bypasses achieve a lower final IOP than a single bypass [91]. Consequently, the two-stent iStent inject has largely supplanted the single iStent in clinical use for greater efficacy [91]. The COMPARE study evaluated the efficacy of the Hydrus and the iStent inject using two-year outcomes. This randomized clinical trial showed that at 24 months, a significantly greater proportion of patients in the Hydrus group achieved a ≥20% IOP reduction without medication (77.3% vs. 57.8%; *p* = 0.02), and more patients in the Hydrus group were medication-free compared to the iStent inject group (84.0% vs. 67.0%; *p* = 0.03), indicating superior efficacy of the Hydrus in reducing both IOP and medication burden [73]. Hence, this suggests that when choosing a trabecular stent, the larger coverage of Hydrus can translate to better efficacy [73]. Analogous comparative studies have been performed between KDB and iStent which have been indicative of KDB’s slight advantage over a single iStent in IOP reduction [31]. However, in a meta-analysis of KDB vs. iStent, the mean change in VA did not differ between groups, indicating that both MIGS approaches preserved vision equally well [92]. Separate trials demonstrate that mean visual field indices are relatively stable for both KDB and iStent, although studies directly comparing visual field indices in KBD vs. iStent are limited [93,94]. Multiple studies demonstrate that both Trabectome and KDB goniotomy have shown no significant change in VA at 12 months compared to baseline [92]. In addition to performing comparisons between MIGS techniques, these studies have served as evidence that although MIGS approaches by themselves do not enhance vision, their safety profile prevents vision degradation and allows for VA maintenance [92].

In addition to RCTs, large real-world series have compared MIGS outcomes. The IRIS Registry and other databases have been mined to compare need for reoperation: one analysis found that Hydrus-implanted eyes had lower rates of subsequent glaucoma surgery at 2 years than those with iStents, hinting at a possible efficacy edge [72]. However, such retrospective comparisons must account for case selection. Overall, the direct evidence supporting one device over another is nuanced—with strong evidence that multiple stents are better than one, and some evidence that an excisional goniotomy is on par with at least two stents [72]. Hydrus and iStent inject, representing the two leading MIGS implants, have both proven effective relative to controls, and ongoing trials (like the COMPASS-XT and others) will shed more light on comparative performance over longer horizons [72].

Another practical consideration is that combining any MIGS with phacoemulsification yields more IOP reduction than phacoemulsification alone [95]. For instance, iStent with phacoemulsification can yield an additional ~2 mmHg IOP drop [95]. Hydrus with phacoemulsification achieved ~80% of eyes with ≥20% IOP reduction vs. ~50% with phacoemulsification alone at 2 years [72]. Additionally, clinical evidence demonstrates that TM MIGS combined with phacoemulsification consistently shows no significant difference in postoperative VA attributable to the MIGS device compared to phacoemulsification alone [96]. The HORIZON Trial (556 patients) is one such study, comparing phacoemulsification with a Hydrus stent to phacoemulsification across 5 years [97]. The HORIZON trial indicates that postoperative VA changes were driven by cataract removal with no between-group VA disparity for Hydrus [97]. Comparably, the US iStent Inject Pivotal Trial (505 patients) performed the same comparison but substituted the Hydrus stent with the iStent inject over a 24-month period [98]. It met its primary 24-month endpoint with 75.8% vs. 61.9% achieving ≥20% unmedicated IOP reduction, and demonstrated a mean unmedicated IOP ~2 mmHg lower in the stent group (15.3 vs. 17.0 mmHg) [98]. This trial achieved similar vision improvement from cataract surgery without vision loss associated with stent usage [98].

MIGS procedures do not reverse existing glaucomatous field loss, but aim to slow the rate of visual field progression by lowering IOP. In the short term (1–2 years), VF outcomes after MIGS generally remain stable relative to pre-op [87,99]. Since mild-to-moderate glaucoma progression is slow, brief trials often show no significant VF change in MIGS or control groups [87,99]. For instance, in the two-year iStent inject trial, both groups had statistically unchanged mean deviation (MD) values over 24 months—indicating no rapid VF deterioration in that time frame, and the MIGS intervention did not produce any deleterious effect on the visual field [87,99]. This is expected, as an appropriately performed MIGS should not damage intraocular structures that affect the visual field [87,99].

### Key Trials and Comparative Studies

A 2-year multicenter study by Holmes et al. found that phacoemulsification and Hydrus and phacoemulsification and iStent inject provided comparable mean IOP reduction, with no statistically significant difference over 24 months [72]. Both groups achieved IOP in the mid-teens with about 70–80% reduction in medication use, and with similar safety profiles [72]. This suggests the Hydrus and iStent inject have a similar range for overall efficacy in mild–moderate OAG; both outperformed historical phacoemulsification-alone results [72]. An indirect comparative trend seen in some analyses is that Hydrus may have an edge in medication reduction [72]. In Holmes et al., at 12 months 94% of Hydrus eyes had ≥20% IOP reduction on the same or fewer meds vs. 73% of iStent inject eyes (*p* < 0.05), though by 24 months this difference was no longer significant [72]. Ongoing follow-up and larger comparative cohorts will further clarify if one device yields more robust long-term control [72].

For Kahook Dual Blade vs. iStent, multiple studies indicate that excisional goniotomy can achieve slightly better IOP outcomes than a single trabecular stent [100]. In one 12-month comparative study of combined phacoemulsification surgeries, phacoemulsification and KDB lowered IOP by ~5.0 mmHg versus ~2.3 mmHg with phacoemulsification and iStent (*p* < 0.001) [100]. A greater proportion of KDB eyes reached ≥20% IOP reduction (64% vs. 42%) [100]. Medication reduction was similar between these two groups [100]. A systematic review and meta-analysis analogously found higher surgical success rates (generally defined as ≥20% IOP reduction) with KDB goniotomy compared to iStent when combined with cataract surgery [92]. By 12 months, eyes treated with KDB were more likely to be at target IOP without medications; the meta-analysis reported an odds ratio indicating ~1.5–2-fold higher odds of success with phaco + KDB versus phaco + single iStent [92]. However, it also noted that the iStent inject achieved IOP lowering comparable to KDB in the few studies available suggesting that single-mechanism MIGS can be compensated by using multiple stents [92]. The safety profiles in these comparisons were equivalent with transient hyphemas, and mild inflammation occurred at similar, low rates in KDB and iStent eyes [92].

Additionally, studies have compared GATT with suture and TRAB360/OMNI with a microcatheter and with KDB. In general, studies show that all angle surgeries that cut through Schlemm’s canal circumferentially have comparable short-term outcomes [32]. For example, Hirabayashi et al. compared KDB goniotomy to 360° trabeculotomy and found both achieved about a 30% IOP decrease and high (≈82–85%) success rates according to ≥20% IOP reduction criteria at 6 months [32]. The choice between using a blade (KDB) vs. a suture or microcatheter (GATT/Trab360) may come down to surgeon preference; the data do not clearly favor one over the other for IOP lowering, although 360° trabeculotomy might achieve slightly lower absolute IOP in cases of very high pre-op pressure [32]. A randomized trial in China found that 360° canal incisions achieved lower IOP than 120° incisions, supporting the logic of complete trabecular removal when possible [101]. Correspondingly, efficacy rankings often reflect the extent of outflow enhancement [32].

MIGS techniques have also been evaluated against more established glaucoma treatments. For instance, the prospective study by Hengerer et al. found that iStent Trabecular Micro-Bypass allowed for a statistically significant mean medication reduction of 57.0 to 69.0% in eyes undergoing combined cataract surgery and iStent inject, and 62.1 to 76.2% in standalone eyes receiving only iStent inject (*p* < 0.001), effectively showing that MIGS alone can substitute for at least one medication [88]. A similar study with iStent implantation at 4 years demonstrated a significant reduction in mean number of medications [102]. While large RCTs have not directly pitted a trabecular MIGS against a first-line laser like selective laser trabeculoplasty (SLT), some insights can be drawn. The LiGHT trial proved SLT to be more effective than drops in preventing VF progression, and MIGS achieved IOP levels on par with SLT outcomes in many patients [103]. In practice, surgeons might choose SLT vs. MIGS based on cataract status and patient preference; both aim to reduce drop burden with minimal risk [103].

Currently, comparisons between MIGS and contemporary, well-functioning trabeculectomy demonstrate superior IOP reduction at ~50% or more [104]. For example, the Tube vs. Trabeculectomy (TVT) study reported mean IOP ~12 mmHg at 5 years after trabeculectomy with MMC [104]. No trabecular MIGS can reliably parallel this IOP reduction as most MIGS are limited by episcleral venous pressure and outflow resistance beyond Schlemm’s canal [105]. The IOP floor with trabecular outflow MIGS is typically the low-teens; achieving IOP in single digits usually requires a subconjunctival pathway or cyclodestruction [105]. Studies comparing MIGS to trabeculectomy are few, but one retrospective matched series found trabeculectomy achieved about 4 mmHg lower IOP than Kahook goniotomy at 2 years, although with more complications [32]. These differences underscore that while MIGS narrow the gap, filtration surgery is still more potent for IOP lowering [32].

However, MIGS vs. trabeculectomy must be weighed in the context of safety. This safety profile has driven adoption of MIGS earlier in the treatment algorithm, with trabeculectomy or tube shunts reserved for when MIGS approaches fail to meet the target [106]. Some surgeons have even explored MIGS in more advanced patients to temporize or as a supplement to reduce risk, but for truly severe glaucoma, traditional surgery is often eventually needed [106]. On the other hand, a proportion of patients who undergo MIGS never need a trabeculectomy thereafter. In HORIZON, the risk of needing future incisional glaucoma surgery in patients with mild to moderate open-angle glaucoma was significantly reduced by MIGS: by 5 years, only 2.5% of Hydrus eyes required a trabeculectomy/tube, vs. 6.4% of the cataract-only eyes [107]. Thus, while MIGS may not lower pressure as significantly as traditional methods, they do provide benefit in delay or obviate the need for more invasive surgery in many cases [107].

## 6. Medication Elimination

The reduction in medication burden is a key measure of efficacy as it improves patient quality of life and medication adherence [107]. Across trials, MIGS reduced medication usage significantly compared to control, with 66–84% of patients requiring no medications at 1–5 years post-op [107]. One systematic review concluded that on average, MIGS patients have a 60% reduction in medication count after surgery [108]. Practically, adding a MIGS can often negate the need for at least one glaucoma drug [108].

It is also useful to consider the IOP levels achieved with MIGS compared with medications. A prospective randomized controlled trial reported that standalone iStent implantation produced IOP lowering comparable to that achieved with topical prostaglandin therapy, with both groups achieving approximately 20% IOP reductions [109]. For example, in HORIZON the Hydrus group’s mean IOP (~16 mmHg on no meds) had similar outcomes on a prostaglandin and beta-blocker regimen without persistent medication to stay in the high teens [107]. Importantly, however, MIGS and medications are not mutually exclusive—they are often complementary [107]. After MIGS, some patients still require one or more drops to reach target IOP, but typically at a lower burden than before surgery [107].

## 7. Complications and Safety Profile

Although TM MIGS have gained traction as a safer, less invasive alternative to traditional glaucoma surgeries, these procedures still pose risks that can impact prognosis and recovery [17,107]. Common and often transient postoperative complications associated with TM MIGS include hyphema that can occur due to disruption of Schlemm’s canal (prevalence 1–30% in trabeculotomy, typically self-limiting, associated with postoperative IOP < 10 mmHg and advanced age), corneal edema (between 2 and 6% prevalence, risk increased with endothelial compromise), and anterior chamber inflammation (up to 12.5% prevalence, where past ocular surgeries and pre-existing inflammatory eye disease increase risk) [83,110]. Long-term complications can occur with MIGS implants like iStent, including stent obstruction and malposition, which may require correction with laser therapy or surgery [106]. Rare but serious complications (<1% incidence) include suprachoroidal hemorrhage (associated with high-risk glaucoma features, hypertension, anticoagulation, high myopia), endophthalmitis (risk factors include pre-existing inflammation, prior ocular surgery, immunocompromised status), and, exceptionally rare, retinal or choroidal detachment after Sinskey microhook ab interno trabeculotomy with cataract surgery, potentially linked to hypotony following certain procedures [83,110,111].

### 7.1. Comparative Trials and Observational Studies on MIGS Safety

TM MIGS approaches vary by design and mechanism of action, leading to distinct safety profiles. Several studies have compared safety outcomes of TM MIGS devices, with current conclusions highlighting the importance of implementing patient-specific risk factors when choosing a MIGS procedure, and the need for further randomized-control studies to verify comparative safety outcomes between MIGS approaches [18]. Major trials are summarized in Table 3.

In one meta-analysis comparing Hydrus Microstent and iStent safety, the total complication rates were similar [26]. However, the Hydrus Microstent was associated with a higher rate of peripheral anterior synechiae, and a lower risk for malpositioning and obstruction events [26]. Implantation of iStents was associated with higher rates of hyphema and malpositioning complications [26]. An additional observational study involving 24-month follow-up for each device found that the Hydrus Microstent had a higher rate of hyphema (40%), peripheral anterior synechiae (20%), corneal edema (10%), and a spontaneous case of self-resolving hypotony (3.3%) [57]. Meanwhile, iStent devices experienced a higher rate of IOP spikes measuring at least 10 mmHg over baseline (6.7% in Hydrus, 11.4% in iStent), which could heighten postoperative complication risk for severe glaucoma cases [57].

Another 12-month retrospective study comparing Kahook Dual Blade (KDB) goniotomy and iStent inject displayed favorable safety profiles for both, with hyphema as the most common complication [112]. Specifically, the iStent group had a hyphema rate of 2.3%, and the KDB group had a rate of 16.3% [112]. KDB goniotomy reported slightly higher hyphema rates and one case of hypotony [112]. In contrast, the iStent inject presented with cases of malpositioning [112]. A further retrospective study comparing the iStent inject and KDB found similar postoperative adverse (IOP spikes, anterior chamber inflammation, corneal edema) outcome rates between the devices [112].

GATT and Trab360 were shown to have heightened efficacy compared to Trabectome, but generally had higher events of hyphema postoperatively [113]. A later study compared Trabectome to KDB, revealing KDB to have lower hyphema rates than the Trabectome [28]. Despite its benefits of having lower rate of hyphema compared to GATT, a meta-analysis comparing MIGS techniques demonstrated that Trabectome was associated with higher rates of IOP spikes compared to the Hydrus Microstent, iStent, and the non-TM MIGS CyPass Micro-Stent [18].

TM MIGS approaches have also been compared to non-TM approaches, although limited studies exist. The CyPass Micro-Stent, a suprachoroidal space device, was associated with a higher frequency of postoperative IOP fluctuations, including both IOP spikes and transient hypotony [114]. One study indicated that CyPass had early safety advantages but raised significant long-term concerns in the form of endothelial cell loss, ultimately leading to its market withdrawal in 2018 [115]. The importance of comparative safety assessment was further highlighted in a study that examined postoperative corneal endothelial changes amongst MIGS devices, revealing that of the three devices examined, iStent demonstrated the least disruption to the corneal endothelium, Hydrus Microstent showed moderate endothelial loss due to suspected increased surgical trauma, and CyPass had the most significant endothelial disruption [116].

### 7.2. Safety Comparison of MIGS with Traditional Glaucoma Surgeries and Medical Management

When compared to conventional glaucoma surgeries such as trabeculectomy, glaucoma drainage devices, canaloplasty, and sclerotomy, MIGS have been shown to have reduced total complication rates [117]. Conventional glaucoma surgeries involve more intraocular tissue manipulation, posing higher rates and risks of complications, such as hypotony, fibrosis and bleb-related infections [114,117]. These complications often require intensive management and follow-up to maintain long-term efficacy and avoid vision loss [106,114]. Due to their less invasive nature, MIGS approaches benefit from reductions in severe complications, having significantly reduced rates of bleb-formation infections, hypotony, and endophthalmitis [118]. MIGS has also been shown to have less frequent rates of common complications, such as hyphema, IOP spikes, and anterior chamber inflammation [18,114]. When these complications do arise in MIGS, they are generally more mild and transient [106,114]. Overall, MIGS has emerged as a key tool in treating mild-to-moderate glaucoma while having a significantly reduced complication risk than the more conventional glaucoma surgeries [117,119].

In the context of pharmacotherapy, while MIGS has a slightly higher likelihood of procedure-related complications, it holds an advantage in the long term due to the higher risk of medication-induced ocular surface disease over time [120]. Both MIGS and pharmacotherapy maintain high safety profiles, but MIGS is typically employed when pharmacotherapy has failed, proven inadequate or fraught with side effects [120]. More comparative studies are needed to evaluate the safety outcomes between MIGS and glaucoma pharmacotherapy.

## 8. Factors Influencing Surgical Success

Multiple factors determine the success of TM MIGS, including the surgical technique and choice of device or procedure. Each of these factors can impact IOP outcomes and the long-term efficacy of the surgery. Technical considerations for TM-based MIGS are summarized in Table 4.

### 8.1. Surgeon Experience and Technique

The outcome of MIGS is highly dependent on the quality of the surgical technique. Surgeon proficiency is a significant determinant of MIGS outcomes, as demonstrated in the literature, which reveals higher initial failure rates that decrease with accumulation of experience [95,121]. Both Trabectome and GATT have been associated with increased efficiency and IOP reduction following surgeons’ first 20–30 cases in studies demonstrating improved success rates with repetition [95,121]. Suboptimal execution compromises patient safety and efficacy of the procedure such as in excess incision depth and iStent misplacement [95,121]. Conversely, dynamic patient head positioning, high-magnification and precise microscope focus to improve angle view can optimize patient outcomes [95,121]. Viscoelastic injection to improve outflow including in adjunctive vasodilation and then meticulous post-operative removal to prevent IOP spiking can further improve operation success rates [95,121]. Many training courses and wet labs are now available for MIGS to augment learning. Thus, surgeon experience directly correlates with MIGS success, as evidenced by lower failure rates and reoperation rates in high-volume MIGS surgeons’ reports [95,121].

### 8.2. Device Selection

When patients require more classes of glaucoma medication, a surgeon might choose a more aggressive MIGS. For example, Hydrus can be selected over iStent inject to maximize efficacy, based on data that Hydrus provides greater IOP reduction, though GATT/OMNI may also be chosen when a stent might not be enough [74]. Notably, the COMPARE study’s findings that Hydrus had better outcomes than two iStents have influenced some surgeons to favor Hydrus when possible for stand-alone glaucoma [98]. When only a mild reduction is needed, single iStent or ABiC suffice with less intervention [98]. Additionally, though all MIGS approaches are safe, contraindications exist. For example, if a patient is prescribed anticoagulants and cannot discontinue, a stent (iStent/Hydrus) might be a safer and less invasive option than GATT [122]. However, if a patient has corneal endothelial compromise (e.g., Fuchs dystrophy), clinicians may avoid devices that stay in the angle purely out of caution [116]. In such a case, a KDB may be chosen to avoid long-term implant-contact risk [116].

Additionally, a patient’s anatomy might favor a particular device. In young patients with a relatively large Schlemm’s canal, a GATT or Hydrus is preferred, while a very small eye or narrow angle might be more amenable to a KDB procedure [93,123]. If there is focal scarring of TM, the surgeon might place a stent away from that area or do a goniotomy to excise it [93,123].

A pragmatic point is that surgeon familiarity significantly influences MIGS device selection and patient outcomes. Consequently, expertly performed procedures may yield better results than less proficiently executed alternatives, even while the latter is advantageous in trials [95,121]. Although device choice can reflect surgeon expertise, selection increasingly tends towards evidence-based selection, matching specific devices to patient needs [95,121]. For example, Hydrus can be leveraged to reduce medication burden and OMNI for comprehensive outflow treatment [107]. However, cost and device availability may limit a surgeon’s ability to tailor a technique to patient needs [95,121].

Alignment between procedural outcomes and patient goals also determines which device should be utilized. For instance, when one device reduces medication burden entirely while another is only partial, the former may be favorable [107]. However, the latter may be used if the patient favors absolute IOP reduction over freedom from medication burden [104]. Safety is another valuable consideration in this regard [117]. It should be noted, however, that another MIGS device may be incorporated to rescue if the previous fails [107]. When sequential MIGS can be attempted, each additional procedure can yield incremental benefit with still a safer profile than a single trabeculectomy in some cases [107].

## 9. Discussion

### 9.1. Controversies and Debates

There is ongoing discussion about which TM MIGS technique yields the best outcomes, reflecting debates on surgical approach and mechanism [73]. TM MIGS share a common goal of enhancing aqueous outflow through Schlemm’s canal, but they achieve this via distinct strategies: stenting the canal, cutting or ablating the trabecular meshwork, or viscodilating Schlemm’s canal [73]. It remains uncertain which approach (bypass vs. excision vs. dilation) is most effective and durable, and this is a matter of debate among surgeons [73]. Advocates of stenting argue that implants scaffold the canal and provide a permanent bypass through the highest-resistance segment of the outflow pathway [73]. In particular, the Hydrus Microstent, which dilates ~90° of the canal, has shown a significant sustained IOP reduction in clinical trials, supporting the concept of stenting and canal dilatation to achieve lasting benefit [73]. Conversely, surgeons favoring excisional goniotomy note that removing or cleaving the TM opens a broad swath of access to Schlemm’s canal without leaving a foreign body, potentially allowing for more fluid percolation into all collector channels [73]. Another point debated by surgeons derives from evidence that suggests increasing the extent of trabecular removal beyond a threshold yields diminishing returns in outflow facilities [73]. For instance, one hydrodynamic study showed less than a 10% additional outflow facility gain when extending a trabeculotomy beyond an initial opening, implying that smaller openings might suffice in many cases [73]. Hence making the optimal extent of angle treatment contentious: for example, whether a 360° trabeculotomy (GATT) confers meaningful advantage over a 90–120° goniotomy [73]. Clinically, a larger treated area often comes at the cost of more intraoperative bleeding, and surgeons must balance this trade-off between maximizing outflow versus minimizing complications [73]. Other concerns presently lacking clarity include how to perform MIGS optimally, individualize MIGS device selection, and identify ideal candidates for MIGS procedures [73]. This is due to variability in patient outcomes arising from differences in surgeon preference and experience, patient anatomy and performance when using intraoperative gonioscopic indicators [73].

### 9.2. Gaps in Research

Despite the rapid adoption of TM MIGS and expanding clinical experience, there remain significant gaps in research that impede consensus [107]. One major gap is the lack of long-term outcome data for most MIGS procedures [107]. Modern MIGS evidence is less than a decade old, and robust ≥5-year data are only now emerging for a few devices such as the Hydrus Microstent with five-year trial results [107]. Many studies report only 1–2 year follow-up, which may not capture late failures or potential rare late complications [107]. Indeed, questions persist about the durability of MIGS IOP lowering effects [107]. In a primate study, an experimentally created trabeculotomy opening was almost completely sealed by regenerated tissue within ~6 months, and the IOP-lowering effect was lost as scar tissue obliterated the canal cleft [35]. It is unknown whether a similar fibrocellular closure happens in human eyes to a significant degree, and longitudinal data is necessary to understanding MIGS’ true longevity [35].

Another critical gap is the paucity of head-to-head trials and comparative studies to guide choice among the various MIGS options [17,18,104]. The lack of such data makes it difficult to rank MIGS by efficacy or safety, or to tailor the MIGS approach to patient subtypes [17,18,104]. Most comparative data is limited to small retrospective series or industry-sponsored non-inferiority trials typically comparing MIGS combined with phacoemulsification to phacoemulsification alone [107]. A related issue is the heterogeneity in outcomes and endpoints across studies, as one study’s definition of success (e.g., % IOP reduction, or IOP ≤ 18 mmHg on meds) may differ from another’s, and follow-up durations vary, complicating inter-study comparability [18]. Glaucoma research would benefit from standardized outcome measures and multicenter trials that directly compare multiple MIGS approaches in parallel [104]. Additionally, health-economic outcomes remain understudied; it is still unclear if MIGS approaches are cost-effective in the disease life course, since device costs are high and robust analyses weighing these costs against savings from reduced medication burden or avoided surgeries are scarce [56].

There are also gaps in understanding the mechanistic and histopathological changes induced by MIGS. MIGS by design aims to restore physiologic outflow, yet the precise tissue response and remodeling that occur after Schlemm’s canal-based procedures are not well-elucidated [16,21]. The wound-healing response in the angle is particularly important; unlike trabeculectomy, which intentionally creates a filtering bleb and is modulated by antimetabolites, MIGS heal mostly below the clinical radar [2,3,52]. Without detailed pathological studies, surgeons are left to infer from clinical observations [2,3,52]. This is a significant gap, because a better understanding of MIGS failure modes could lead to preventative measures, such as anti-fibrotic adjuncts or modified device designs [95,121].

Finally, there is a recognized knowledge gap regarding how patient factors and surgical technique modulate MIGS outcomes, underscoring the need for more nuanced research and guidelines. Clinicians currently have limited tools to predict who will respond best to a given MIGS. As noted, MIGS efficacy may hinge on a patient’s individual outflow system anatomy and function [21,118]. Preliminary work using anterior segment OCT angiography has shown that eyes with certain patterns of deep episcleral vasculature achieve greater IOP reduction after TM MIGS, suggesting that quantifiable markers might forecast surgical success [21,118]. Validating such biomarkers in larger studies is an important research goal, as it could allow surgeons to better match patients with the procedure most likely to benefit them, thereby developing preoperative assessment methods [21,118]. Moreover, subgroup analyses in MIGS studies are needed to determine if outcomes differ by glaucoma subtype (e.g., primary open-angle vs. pseudoexfoliative glaucoma), by lens status (phakic vs. pseudophakic), or by prior treatments [21,118]. The influence of surgeon technique also warrants systematic study [21,118]. While it is accepted that there is a learning curve, there is little published data quantifying how much outcomes improve with surgical experience or which technical maneuvers yield statistically better results [21,118,121]. In addition, standardizing MIGS surgical protocols where possible may reduce inter-study variability, but research is needed to identify best practices [121]. By filling these knowledge gaps, clinicians will be better equipped to judiciously deploy TM MIGS procedures and refine them further [121]. Continued research will help resolve current controversies and ensure that the enthusiasm for MIGS is supported by solid evidence and optimized patient outcomes [121].

## 10. Conclusions

TM MIGS shows promise as a therapeutic option for meaningful IOP reduction and reduced medication burden in patients with mild to moderate OAG who are not achieving IOP control with other interventions or who are undergoing cataract surgery. It remains unclear whether one TM MIGS technique is superior, as outcomes are influenced by the extent of trabecular intervention, distal outflow anatomy, surgeon experience, and patient factors. Although TM MIGS uptake continues to increase, gaps remain, including a lack of long-term outcomes, larger studies to validate quantitative markers for surgical success, subgroup analyses, and the influence of surgeon technique on TM MIGS outcomes. Future research should explore standardized outcomes in TM MIGS and compare techniques to enable integration into glaucoma care.

## Figures and Tables

**Table 1:** Comparison of TM-based MIGS devices.

Device/Technique	Pros/Efficacy	Cons/Safety	Technical Considerations
iStent (trabecular micro-bypass stent)	Significant IOP reduction and reduced medication need when combined with cataract surgery Very high safety profile; minimally invasive with rapid recovery	Modest IOP lowering as a standalone (often insufficient for advanced glaucoma) Placement is delicate; malposition can lead to suboptimal IOP control	Inserted ab interno into Schlemm’s canal under gonioscopy via a clear corneal incision Single tiny titanium stent (0.3 mm), making it difficult to visualize and implant, requiring surgical experience
iStent inject (two second-generation trabecular stents)	Dual stents increase outflow through two channels, yielding greater IOP reduction than a single iStent Maintains excellent safety; no significant added risk over single stent	Similar limitations as iStent (dependence on collector channel patency; moderate IOP reduction ceiling) Requires open angle; not for angle-closure or extensive synechiae	Uses preloaded injector to deploy two stents ~2–3 clock hours apart FDA-approved in 2018; typically combined with cataract surgery (US labeling)
Hydrus Microstent (Schlemm’s canal scaffold)	Larger IOP reduction and med reduction vs. iStent inject in head-to-head trials “Trimodal” action: TM bypass + canal dilation + coverage of ~3 clock hours (potentially accessing multiple collector channels) 5-year data show sustained IOP control without added safety issues	Slightly more invasive insertion (8 mm stent), so risk of malposition or protrusion if not placed fully Theoretically could touch cornea or angle structures if mis-positioned (though 5-year study showed no corneal endothelial harm)	Nitinol stent delivered via preloaded injector, typically done with cataract surgery Spans 90° of the canal; ensure full insertion to avoid the stent end protruding. FDA-approved in 2018
Trabectome (electrocautery trabeculotomy)	Can achieve substantial IOP decrease (~30–40%) and medication reduction Favorable safety: minimal incisional trauma, relatively low complication rate (mainly brief hyphema) Versatile: used in phakic or pseudophakic eyes, even in narrow angles or secondary glaucomas	Requires expensive console and handpiece; steep initial learning curve for proper angle approach and ablation. Commonly causes blood reflux from Schlemm’s canal intraoperatively (transient hyphema), which can cloud the view IOP reduction may be insufficient in advanced glaucoma, where outflow pathways beyond TM are damaged.	Ab interno electrocautery that ablates ~60–120° of TM under direct gonioscopic view Footplate protects surrounding tissue; aspiration removes debris FDA-approved in 2004; often combined with phaco for greater effect
TRAB360 (360° trabeculotomy device)	Achieves complete 360° trabeculotomy, maximizing outflow enhancement. Can be used in pediatric and adult glaucoma	Higher hyphema incidence due to extensive TM disruption Technically more demanding than focal goniotomies.	Microcatheter is inserted into Schlemm’s canal and withdrawn to tear TM 360°
Kahook Dual Blade (dual-blade goniotomy)	Excises TM strip with minimal collateral damage, which may reduce fibrosis and enhance long-term patency IOP reductions of around 20% or more in the majority of patients; effective across various OAG types (PXF, pigmentary, steroid-induced) No implanted device and no capital equipment needed (cost-effective per case)	Focal goniotomy (typically 3–4 clock hours) has IOP lowering that may be less than 360° trabeculotomy in some cases Transient hyphema is common (due to unroofing Schlemm’s canal), though usually mild Angle anatomy variants (high insertion iris processes) can make smooth blade passage challenging	Performed ab interno with direct gonioscopy view; the dual blades incise parallel cuts in TM, which allows a strip of TM to be peeled/excised Typically, one quadrant treated per pass; can rotate and repeat to extend goniotomy if needed FDA approved 2015
GATT (360° suture trabeculotomy ab interno)	Achieves near-complete trabeculotomy (360°), often yielding larger IOP drops (30–40%) especially in high-pressure glaucomas Device-independent (uses suture or microcatheter), lowering cost; adaptable if specialized MIGS devices are unavailable Proven highly effective in pediatric and juvenile glaucomas where 360° trabeculotomy addresses the primary outflow block	Almost universal transient hyphema due to extensive Schlemm’s canal opening Technically intricate: threading a catheter or suture 360° in Schlemm’s canal can be challenging, especially if scarring or small canal Not ideal in patients on anticoagulation or bleeding disorders (risk of prolonged hyphema)	Requires an initial goniotomy (small TM opening) to introduce a microcatheter or 5–0 Prolene suture into Schlemm’s canal Under gonioscopic guidance, thread circumferentially and retrieve the lead end. Then, pull through to tear the TM around 360° First described 2014; no implant left in eye
Excimer Laser Trabeculostomy (ELT)	No implant and no significant tissue excision, instead uses “cold” laser energy, causing minimal collateral damage High safety profile: very low risk of hypotony or serious complications; quick recovery Can be easily repeated or combined with cataract surgery; leaves angle structures intact, aside from microperforations	Not widely available in all regions; specialized laser equipment needed. IOP reduction moderate (20–30% range)—may not achieve very low target pressures Limited data in US populations; most studies from Europe with somewhat varied protocols	Performed ab interno under gonioscopic view with a fiber-optic probe that delivers 308 nm excimer laser pulses to TM About 8–10 micro-channels are created over ~90–120° by pulsed ablation of TM, spaced ~500 µm apart Each channel connects AC to Schlemm’s canal, facilitating outflow
Ab Interno Canaloplasty (ABiC)	Restores physiological outflow by dilating existing channels without permanently breaching TM (less risk of scarring than incisional goniotomy) Very safe: minimal tissue removal, typically no significant hyphema since TM is left intact Useful in eyes where a smaller IOP reduction is acceptable and overt trabeculotomy is not desired (e.g., mild OAG, ocular hypertensives)	IOP reduction is limited by leaving TM intact, so efficacy may be less than that of goniotomy (often ~25–30% drop) Requires expensive microcatheter and viscoelastic; technically careful catheterization needed (similar to GATT threading without tearing). Long-term durability unclear; TM might gradually revert if canal narrows again.	After a corneal incision, a small goniotomy or ostomy is made to insert the iTrack illuminated microcatheter into Schlemm’s canal Catheter is threaded 360° and viscoelastic is injected throughout to viscodilate the canal and distal outflow tracts, then catheter is withdrawn No stent is left; TM is not excised

Abbreviations: IOP = intraocular pressure, OAG = open-angle glaucoma, PXF = pseudoexfoliation glaucoma.

**Table 2:** Differences in efficacy of MIGS devices with respect to IOP reduction, visual acuity and visual field. ↓: the down arrow represents “decrease”. →: the horizontal arrow represents a change in IOP value.

MIGS Device	IOP Reduction	Visual Acuity (VA)	Visual Field (VF)
iStent (1st-gen)	~20–25% IOP reduction. In combined cataract cases, mean IOP ↓ ~4 mmHg (~23% from ~17 mmHg baseline). As a standalone, meta-analyses show significant IOP lowering vs. baseline. Typically reduces medication need by ~1 drug.	Generally unchanged. No significant VA loss attributable to stent; VA often improves if combined with cataract surgery (due to cataract removal).	No immediate VF change. Intended to stabilize VF by lowering IOP. Short-term trials show VF preservation consistent with IOP control; no acceleration of field loss noted versus controls in 1–2 year studies.
iStent inject (2nd-gen)	~30–40% IOP reduction. Dual micro-stents achieve greater IOP lowering than a single iStent. Long-term series (7 years) report a ~34–44% IOP reduction (e.g., from ~23.5 mmHg to ~13–15 mmHg) with two stents. Often eliminates 1–2 medications.	Generally unchanged. Maintains VA similarly to cataract-only surgery; no significant difference in VA outcomes between combined phaco + iStent inject vs. phaco alone.	No short-term VF improvement (VF loss is irreversible). Long-term data show low rates of VF progression, e.g., <5% of eyes had clinically significant VF loss over 7 years in one study, suggesting effective VF stabilization.
Hydrus Microstent	~30% IOP reduction (covers ~90° of Schlemm’s canal). In RCTs, combined phaco + Hydrus achieved ~2 mmHg lower IOP than phaco alone at 2 years (unmedicated). IOP lowering is durable; 5-year data show Hydrus maintains significant IOP and medication reduction vs. controls.	Generally unchanged. Similar VA outcomes to cataract surgery alone since the microstent procedure itself is visually non-invasive.	Long-term VF preservation demonstrated. At 5 years, Hydrus combined with cataract showed a slower mean rate of VF loss (−0.26 dB/year) than cataract alone (−0.49 dB/year). Significantly fewer Hydrus-treated eyes had large VF declines, indicating VF stability over time.
Trabectome (ab interno trabeculectomy)	~20–30% IOP reduction. Removes ~120° of trabecular meshwork with electrocautery. Typical outcome: IOP reduction ~5 mmHg (e.g., from ~18 → 13 mmHg) in moderate glaucoma, or ~30% from higher baselines. About one fewer medication on average postoperatively. Long-term studies (up to ~7.5 years) show IOP reduced from ~23 → 16 mmHg (~29% drop) with sustained benefit	Generally unchanged. Trabectome’s ab interno approach spares the conjunctiva and causes only transient postoperative effects (e.g., mild transient vision blur from micro-hyphema in ~6% of cases). Long-term, no significant VA loss; VA is typically stable or improved if combined with cataract.	Stable. No improvement in VF (damage is permanent), but by lowering IOP Trabectome can slow further VF loss. Long-term follow-up indicates maintained VF stability in most patients (e.g., ~60% survival at 90 months by strict success criteria).
TRAB360 (360° ab interno trabeculotomy)	~30–35% IOP reduction. Uses a microcatheter or suture to circumferentially trabeculotomize Schlemm’s canal. Mean IOP drop ~7 mmHg at 12 mo (e.g., 22 → 15 mmHg) in refractory POAG. ~59% of eyes reach ≥20% IOP reduction and <18 mmHg on ≤baseline meds at 1 year. Often reduces medication burden (~0.5–1 fewer).	Generally unchanged. VA typically maintained; significant vision improvement can occur if combined with phaco. No intraocular hardware is left in the eye, and complications (mainly transient hyphema) rarely affect VA long-term.	Stable. By eliminating trabecular resistance, GATT/Trab360 provides substantial IOP control, which translates to VF stabilization in most patients. Approximately 70–85% of eyes avoid further glaucoma progression or surgery at 1–2 years. Longer-term success depends on disease severity; high initial IOP eyes tend to benefit the most (e.g., patients with baseline IOP ≥25 mmHg had ~67% success at 1 year under strict criteria).
GATT (gonioscopy-assisted transluminal trabeculotomy)	~30–50% IOP reduction (especially effective in high-IOP and secondary glaucomas). In POAG, mean IOP drop ~39% at 12 mo (e.g., ~28 → 17 mmHg) with ~1 fewer medication. In eyes with secondary glaucoma (e.g., PXG), IOP reductions ~50%+ have been reported (e.g., 32 → 15 mmHg). High long-term success in many series (≈60–85% of eyes achieve sustained IOP control without needing filtering surgery).	Generally unchanged. GATT is an ab interno procedure (via an existing clear-corneal incision), so it does not induce significant refractive or corneal changes. VA is typically unchanged aside from cataract removal if combined. Mild transient hyphema is common post-op but rarely causes lasting VA issues.	Stable or improved control. GATT can markedly slow glaucoma progression by achieving low-to-mid teens IOP. In case series, VF stability is maintained in the majority of patients after GATT due to the robust IOP reduction. Eyes that achieve low IOP (≤15 mmHg) after GATT tend to have minimal VF progression; one study reported a surgical success rate of 83.7% (IOP from 25.0 → 15.9 mmHg) with corresponding VF stabilization in most cases.
Kahook Dual Blade (KDB goniotomy)	~25–30% IOP reduction. Exercise a strip of TM (~3–4 clock hours). In combined phaco + KDB, mean IOP reduction ~5 mmHg at 12 mo (18.2 → 13.2 mmHg) versus ~2 mmHg with phaco + iStent. KDB lowered IOP significantly more than a single iStent in head-to-head analyses (e.g., 64% vs. 42% of eyes with ≥20% IOP drop). IOP outcomes of KDB are comparable to those of two iStents. Typically reduces medication burden by ~1 medication.	Generally unchanged. KDB goniotomy has a safety profile similar to other ab interno trabeculotomies: transient mild hyphema or inflammation can occur but usually resolves without VA impact. No significant change in VA long-term relative to baseline or to comparative MIGS procedures.	Stable. By enhancing aqueous outflow, KDB helps prevent further VF loss. Clinical studies up to 12–24 mo show no worsening of VF indices; lowering IOP by ~25–30% should confer a reduced risk of progression akin to adding an extra medication. A meta-analysis found higher “surgical success” rates for phaco + KDB vs. phaco + stent procedures, implying effective IOP control that should translate into VF stabilization.
Excimer Laser Trabeculostomy (ELT)	~30% IOP reduction. Creates 8–10 micro-perforations in TM with a 308 nm excimer laser. Typical outcomes: IOP reduction from ~22 → 14–17 mmHg (≈6–8 mmHg drop) sustained long-term. For example, in one 8-year study: ELT-alone group IOP ~22 → 15.9 mmHg; ELT + phaco group 22 → 13.7 mmHg at final follow-up. Medication use is also reduced (often by 1 med) initially, though some patients required meds again over several years.	Generally unchanged. ELT is tissue-sparing (no implant); it has a high safety profile with minimal collateral damage. VA remains stable; when combined with phaco, patients enjoy the normal visual improvement from cataract surgery. No significant corneal or retinal side effects long-term.	Stable. Long-term studies indicate durable IOP control with VF preservation. At 2 years, ELT lowered IOP ~32% (25 → 16.9 mmHg) vs. no change in controls. Extended follow-ups (4–8 years) show maintained mid-teens IOP and low progression rates. VF outcomes are comparable to other MIGS approaches: effective IOP reduction correlates with slowed VF deterioration, and no excess VF loss has been observed in ELT-treated eyes over many years.
Ab Interno Canaloplasty (ABiC)	~35% IOP reduction. Uses an illuminated microcatheter to viscodilate Schlemm’s canal 360° (no stent left in situ). Typical result: IOP reduction from ~20–21 mmHg to ~13–14 mmHg by 12 mo (maintained at 24–36 mo). In a 3-year series, mean IOP was ~13.3 mmHg at 36 mo vs. 20.5 mmHg pre-op (≃35% drop). Medications reduced from ~2.8 to ~1.1 at 1 yr, with most patients on ≤1 med by 3 yrs.	Generally unchanged. ABiC spares the conjunctiva and causes minimal intraocular trauma. Postoperative VA is typically the same as preoperative (aside from any cataract extraction effect). No bleb means no risk of bleb-related vision issues; no significant corneal complications reported.	Stable. By improving outflow facility, ABiC lowers IOP into the low-teens, which should slow glaucoma progression. Three-year studies show ~95% of eyes achieve IOP ≤17 mmHg on reduced meds. Patients tend to maintain their baseline VF status; ABiC’s efficacy in mild–moderate glaucoma suggests it helps prevent VF worsening comparable to other trabecular bypass procedures (with the advantage of an implant-free, viscodilation approach).
Ab-interno needle goniectomy (BANG)	~20% IOP reduction in the short-term. Uses a hypodermic needle to improve aqueous outflow. In an interventional study, mean IOP decrease was from 15.3 to 13.6 mmHg at six months post-op. The mean number of topical anti-glaucoma medication used went from 2.6 to 0.60 at 6 months post-op.	Studies did not report significant decreases in visual acuity 1-year post-BANG. Although transient hyphema is the most common adverse effect, studies did not report long-term impact on visual acuity.	Visual field outcomes were stable. Studies indicated that visual field mean deviation remained stable at 12 months post-op.

**Table 3:** Major MIGS trials.

Name	Study Design	Device	Number of Eyes	Results
The HORIZON Trial (2018)	24 month prospective, multicenter, single-masked, randomized controlled clinical trial (38 sites)	Hydrus Microstent	369 years with Hydrus Microstent; 187 eyes with cataract surgery only	After 2 years, 77.3% of eyes with Hydrus Microstent + cataract surgery had an IOP reduction of ≥20% (no medication) versus 57.8% of the eyes receiving only cataract surgery
iStent inject pivotal trial (2019)	24 month prospective, randomized, single-masked, concurrently controlled, multicenter clinical trial	iStent inject	505 eyes with mild to moderate primary open-angle glaucoma	In the iStent inject + cataract surgery group, 75.8% of eyes had a ≥20% reduction in unmedicated DIOP compared vs. 61.9% of control eyes, with a greater mean IOP reduction (7.0 vs. 5.4 mmHg), higher medication free rates (84% vs. 67% of responders), and a similar safety profile
COMPASS XT Trial (2019)	Prospective, 24-month, randomized, multicenter, controlled trial conducted at 24 US sites	Supraciliary microstent	505 eyes (374 microstent and 131 control) with mild to moderate primary open angle glaucoma	77% of microstent eyes compared to 60% of cataract-only controls had ≥20% unmedicated IOP reduction (mean IOP lowering of −7.4 vs. −5.4 mmHg), and higher medication-free rates (85% vs. 59%) There were no vision-threatening device-related adverse events
COMPARE Study (2020)	Prospective, multicenter, randomized clinical trial	Hydrus iStent inject	152 eyes with open-angle glaucoma	A significantly greater proportion of patients in the Hydrus group achieved a ≥20% IOP reduction without medication (77.3% vs. 57.8%; *p* = 0.02) More patients in the Hydrus group were medication-free compared to the iStent inject group (84.0% vs. 67.0%; *p* = 0.03)
Sato et al. (2021)	12 month prospective, single-center, three-arm randomized trial	Schlemm’s canal suture trabeculotomy (360° vs. 180° vs. <180°)	99 eyes with open-angle glaucoma	Suture trabeculotomy ab interno significantly reduced intraocular pressure and medication use There were no significant differences in efficacy between the 360°, upper-180°, and lower-180° incision groups Hyphema was more common in the 360° group
The GEMINI Study (2022)	24 month prospective, multicenter trial (15 locations)	OMNI Canaloplasty plus trabeculotomy with phacoemulsification	120 eyes with cataract and mild to moderate open-angle glaucoma	OMNI canaloplasty plus trabeculotomy with phacoemulsification decreased mean unmedicated diurnal IOP from 23.8 ± 3.1 to 15.6 ± 4.0 mmHg (−35%), and decreased medications from 1.8 to 0.4 (−80%) 84.2% of eyes had >20% IOP reduction, and 80% of eyes were medication-free Good safety profile

**Table 4:** Technical considerations and limitations for TM-based MIGS.

MIGS Device/Technique	Learning Curve	Device or No Device	Reversibility/Future Options
iStent	Technically challenging due to very small size. Requires precision to insert into Schlemm’s canal.	Involves implanting a device. Some surgeons avoid it due to cost or preference against foreign material.	Does not interfere with future trabeculectomy/tube; stents can be left in place.
iStent inject	Easier than original iStent; widely adopted due to streamlined injector and dual stent deployment.	Device-based; may be preferred by cataract surgeons due to ease during phaco.	Safe to perform future filtering surgery; implant stays in place.
Hydrus	Easier to position than iStent due to larger size, but requires longer incision and more intraocular manipulation.	Device-based; may not be covered in all systems, particularly as a standalone procedure.	Can be left in place; does not preclude future filtering procedures.
Trabectome	Requires coordinated hand and foot control; learning curve for electrocautery and fluid management.	No long-term implant left behind.	TM ablation does not prevent future surgeries, but angle anatomy may be altered.
Kahook Dual Blade (KDB)	Short learning curve; simple drag-and-excise motion. Easy to adopt.	Implant-free; lower cost. Attractive in systems with limited device reimbursement.	Removes a segment of TM; theoretically could affect future filtration but generally does not.
GATT	Technically most demanding. Requires threading a suture or microcatheter 360° through Schlemm’s canal.	No device left behind. Very low cost (just a suture).	Some concern that full circumferential trabeculotomy may lead to canal scarring, though evidence is limited. Filtering surgery still possible.
TRAB360/OMNI	Mechanizes GATT; makes procedure more standardized but still technically involved.	Device is disposable; cost can be limiting in some healthcare systems.	Like GATT, may affect future surgeries if Schlemm’s canal becomes fibrosed, but many proceed successfully.
ELT	Moderate learning curve; laser aiming and probe control needed.	No device; laser-based ablation.	Does not remove tissue; minimal disruption allows compatibility with future surgery.
ABiC	Requires microcatheter navigation and controlled viscodilation. Easier than full GATT but still technically involved.	No implant used.	Preserves TM; excellent compatibility with future MIGS or filtering surgeries.

## Data Availability

All inquiries regarding access to research data should be addressed to the first author.

## Abbreviations

The following abbreviations are used in this manuscript:

AC	Anterior chamber
ABiC	Ab interno canaloplasty
ELT	Excimer laser trabeculostomy
EVP	Episcleral venous pressure
FDA	Food and Drug Administration
GATT	Gonioscopy-assisted transluminal trabeculotomy
IOP	Intraocular pressure
KDB	Kahook Dual Blade
MIBS	Minimally invasive bleb surgery
MIGS	Minimally invasive glaucoma surgery
OAG	Open-angle glaucoma
OCT	Optical coherence tomography
OVD	Ophthalmic viscoelastic device
PXF	Pseudoexfoliation (glaucoma)
RGC	Retinal ganglion cell
TM	Trabecular meshwork
VA	Visual acuity
VF	Visual field
